# Late-life depression and increased risk of dementia: a longitudinal cohort study

**DOI:** 10.1038/s41398-021-01269-y

**Published:** 2021-03-02

**Authors:** M. Ly, H. T. Karim, J. T. Becker, O. L. Lopez, S. J. Anderson, H. J. Aizenstein, C. F. Reynolds, M. D. Zmuda, M. A. Butters

**Affiliations:** 1 Department of Psychiatry, University of Pittsburgh, Pittsburgh, PA USA; 2 Department of Neuroscience, University of Pittsburgh, Pittsburgh, PA USA; 3 Department of Psychology, University of Pittsburgh, Pittsburgh, PA USA; 4 Department of Neurology, University of Pittsburgh, Pittsburgh, PA USA; 5 Department of Biostatistics, University of Pittsburgh, Pittsburgh, PA USA; 6 Department of Bioengineering, University of Pittsburgh, Pittsburgh, PA USA

**Keywords:** Scientific community, Depression

## Abstract

Late-life depression (LLD) is associated with an increased risk of developing dementia; however, it is not known whether individuals with a history of LLD exhibit a more rapid rate of cognitive decline. We aimed to determine whether those with LLD experienced faster cognitive decline compared with never-depressed control (NDC) participants from the community and whether stratification of LLD into early-onset depression (EOD) and late-onset depression (LOD) subtypes revealed differing rates and domain-specific expression of cognitive decline. We conducted a prospective, longitudinal study where 185 participants with LLD (remitted) and 114 NDC were followed for 5 years on average. EOD was defined as having first lifetime depressive episode at <60years and LOD at ≥60years. Every year, participants underwent comprehensive neuropsychological assessment. Composite scores for each cognitive domain were calculated through averaging standardized scores across tests. LLD compared to NDC demonstrated significant baseline impairment but did not decline more rapidly. EOD were significantly impaired in attention/processing speed and global cognitive function at baseline but did not experience more rapid decline as compared to NDC. Those with LOD compared to both NDC and EOD performed worse in all domains at baseline and experienced more rapid decline in verbal skills and delayed memory ability. Our findings suggest that baseline impairment may lower the threshold for those with LLD to develop dementia. EOD and LOD may represent distinct phenotypes of cognitive impairment with differing neural substrates. LOD may represent a distinct phenotype with a more rapid decline in verbal skills and delayed memory.

## Introduction

Late-life depression (LLD) is a leading contributor to psychiatric and medical morbidity and mortality in older adults^1^. Often treatment resistant and recurrent, LLD is associated with highly prevalent cognitive impairment (~50%) that is persistent even after remission of depressive symptoms^2,3^. Specifically, LLD has been associated with a twofold increase in risk of developing multiple types of dementia, including Alzheimer’s and vascular dementia^4,5^. However, it is not clear whether individuals with a history of LLD experience a more rapid rate of cognitive decline in light of their increased risk of developing dementia. Clarification of the rate of cognitive decline in LLD may provide valuable clinical insight into risk stratification and possible prevention of (including timing of intervention for) future dementia.

The heterogeneous and multifactorial etiologies involved present a significant challenge in the process of predicting long-term neurocognitive and other outcomes in the course of LLD^6,7^. Individuals with LLD present with a wide range of neuropathological changes, brain structural abnormalities, and levels of cognitive functioning at baseline and following an episode of depression. It is unclear whether these abnormalities are related to the etiology of LLD or whether they represent the consequences of LLD itself. Clinical attributes, such as depression exposure (length of and number of depressive episodes), education level, and medical comorbidity, are also significant sources of variability. A potential avenue to reduce heterogeneity in LLD is through stratification of LLD into separate phenotypes.

Age of onset of the first depressive episode is highly related to depression exposure and therefore may represent a useful phenotypic distinction. Early-onset depression (EOD) is thought to stem from genetic predisposition and adverse life events, while late-onset depression (LOD) is more associated with the accumulation of vascular burden and other pathologic aging processes in the absence of family history^8,9^. EOD patients may experience cognitive impairment due to longer time in depression or more lifetime depressive episodes, which lead to hippocampal atrophy, increased allostatic load, and decreased brain reserve. In contrast, cognitive impairment in LOD patients may result directly from vascular and neurodegenerative risk factors, which may also be the major precipitant of the depressive episode^8,9^. If EOD and LOD represent distinct phenotypes of LLD, it is critical to investigate whether cognitive trajectories differ between the two groups over time.

Previous investigations of the long-term cognitive outcomes in LLD have largely been cross-sectional, while longitudinal studies often did not exceed 5 years in duration, thereby limiting their ability to delineate longer neurocognitive trajectories. Other limitations of prior studies include small sample sizes, reliance on imprecise cognitive screening measures and limited use of highly replicable broad-based neurocognitive batteries, and lack of measurement and/or statistical control of baseline cognitive functioning. In addition, many studies did not differentiate between EOD and LOD in reporting LLD subgroups. One study did stratify outcomes in EOD and LOD patients using a robust longitudinal design, but obtained baseline neuropsychological measurements while patients were depressed, which potentially confounds the interpretation of cognitive performance^10^. They found that individuals with LLD exhibited greater cognitive impairment at baseline and greater subsequent decline, but also found that EOD had the greatest impairment with the greatest declines while LOD exhibited less impairment and slower decline but still more than control participants.

The primary purpose of this longitudinal study was to determine whether individuals with a history of LLD experience more rapid cognitive decline than those without a depression history. Participants with a history of LLD and never-depressed control (NDC) participants underwent annual neuropsychological assessments for up to 10 years. All baseline assessments and most subsequent assessments were conducted while LLD participants were in a state of remission, but we controlled for any depressive symptoms regardless. Baseline cognitive performance and rate of cognitive decline were compared between the LLD and NDC groups. We hypothesized that individuals with a history of LLD would have more cognitive impairments at baseline and exhibit more rapid decline over time in multiple domains of cognitive performance compared with the NDC group. We also investigated whether dichotomizing the LLD group into EOD and LOD phenotypes revealed differing rates of cognitive decline. The analyses were exploratory because there are competing hypotheses that predict poor performance from each of these two subgroups. Those with EOD may perform poorly due to multiple previous depressive episodes involving neurotoxic processes. On the other hand, individuals with LOD may be in the prodromal stage of a neurodegenerative disorder. Each of these scenarios may lead to impaired cognitive performance.

## Methods

### Participants

Participants with LLD were recruited from the University of Pittsburgh Late-Life Depression Prevention and Treatment Center (*N* = 185), and NDC (*N* = 114) were recruited from the local Pittsburgh community. Participants with LLD were treatment-seeking individuals who were recruited into this study following successful treatment. Recruitment occurred on a rolling basis which allowed for more data acquisition and longer follow-up from participants recruited in the early years of the study. Inclusion criteria for LLD participants stipulated age 60 or older at baseline visit, meeting *Diagnostic and Statistical Manual of Mental Disorders, Fourth Edition* (DSM-IV) criteria for unipolar major depression, English language fluency, and visual and auditory acuity adequate to undergo neuropsychological assessment. Exclusion criteria included major unstable medical illness, diagnosis of psychiatric disorders other than unipolar major depression or anxiety disorders (except for generalized anxiety disorder (GAD) or specific phobia), neurologic disorders or injuries with direct effects on cognitive functioning, and clinical diagnosis of dementia. While patients could have an anxiety disorder diagnosis (limited to GAD or specific phobia), late-life major depressive disorder (MDD) symptoms and disorder had to be most prominent for a participant to be included. Control participants were recruited from the Pittsburgh community and met the same inclusion and exclusion criteria, with the exception that they had no lifetime history of any psychiatric disorder. Lifetime antidepressant exposure was reported in 93.5% (*N* = 173) of individuals with history of LLD and 9.6% (*N* = 11) of NDCs for indications other than depression. Over the duration of this study, ~70% of the LLD group and ~10% of the NDC group were taking antidepressant medication. LLD participants were further categorized into EOD (*N* = 85) and LOD subgroups (*N* = 100), with early-onset defined as having lifetime depressive episode at age 59 or younger and late-onset defined as first lifetime depressive episode at age 60 or older. All participants provided informed consent under a protocol approved by the University of Pittsburgh Institutional Review Board.

### Procedures

At baseline and annual follow-up visits, participants were assessed for depressive symptoms (17-item Hamilton Depression Rating Scale, HDRS-17), medical comorbidity (Cumulative Illness Rating Scale for Geriatrics, CIRS-G), and cardiovascular risk factor status (CVRF; risk factors derived from Probability of Stroke Risk Profile from the Framingham Heart Study). At every follow-up visit, in order to ensure that cognitive assessments were conducted during a period of remission or reduced depressive symptoms, we aimed to administer the neuropsychological test battery only if participants’ HDRS-17 score was ≤10. Participants with HDRS-17 score >10 were referred to treatment, with study visits postponed until symptomatic improvement had occurred (postponed by up to 3 months as needed). All depressed participants were referred to one of several ongoing depression intervention studies; although the pharmacotherapy varied, all studies conducted within the University of Pittsburgh Late-Life Depression Prevention and Treatment Center entailed protocolized pharmacologic intervention. However, some individuals had persistent depression that did not remit at one or more subsequent visits and ultimately were assessed. Approximately 21% of visits [376 out of 1774 total visits across all participants] took place with a participant with an HDRS-17 score >7 (criteria for remission) and 9% of visits [159 out of 1774 total visits across all participants] took place with a participant with an HDRS-17 score >10. On average, participants had 1.3 (SD 1.6) visits with a HDRS-17 > 7, and 0.5 (SD 0.9) visits with a HDRS-17 > 10, reflecting their persistent, low-level depressive state.

The neuropsychological battery used in this study has been well-validated in assessing cognitive function across multiple domains in older adults, as detailed in our prior work^11,12^. As in our previous work, the raw scores of each neuropsychological test were converted to standard scores using the distribution of the NDC group. Composite scores for each cognitive domain (attention/processing speed, visuospatial ability, verbal ability, executive functioning, and delayed memory) were then calculated by averaging the standard scores across tests in Supplemental Table 1. Selection of cognitive domains was guided by factor analysis, conceptual groupings, and Cronbach’s alphas. The Cronbach’s standardized alpha values ranged from 0.55–0.75.

### Statistical Analysis

Prior to any analysis, we examined data distributions to assess normality and the presence of outliers. We calculated descriptive statistics for baseline demographics and clinical measures of the NDC and LLD groups, using *t* tests to test for group differences on the continuous variables and chi-square tests for categorical variables.

We plotted cognitive domains over time for both groups to examine individual domain trajectories as well as the mean (and standard error) for each group. After reviewing the graphs, we chose to use 10 years of data in all analyses to maximize clinical relevance and to minimize bias estimates due to drop off in sample size and increased variability after 10 years. Baseline date and yearly visit dates determined the time variable for all analyses. In SAS, we employed a mixed-effect models repeated measures approach to compare domain trajectories and to test for group, time, and group by time differences^13^ with an unstructured covariance matrix. When fitting the models, we controlled for baseline cognitive domain scores since the two groups differed at baseline. Baseline age, medical comorbidity, education, and sex were also included in the model as covariates since these are known to affect cognitive function. Models first considered quadratic effects to test for nonlinear trajectories. When the quadratic component was not significant, we moved to a linear model. A best fit model was determined by comparing Bayesian Information Criterion values between models. The models were fit assuming that the missingness over time was at random or completely at random^14^ and utilized pairwise deletion for incomplete data.

### Role of the funding source

The funding sources had no role in the design and conduct of the study, data collection, data analysis, data interpretation, or writing and review of the manuscript. The corresponding author had full access to all data in this study and had final responsibility for submission of the manuscript for publication.

## Results

The demographic and clinical characteristics of the study participants comparing LLD and NDC are displayed in Supplemental Table 2. The LLD group compared to NDC was older, had a greater percentage of female participants, and had greater medical comorbidity and vascular risk factors as determined by the CIRS-G and CVRF, respectively. Mean length of follow-up did not differ between NDC (5.7 years, 1–14.8 years) and LLD (5.7 years, 0.9–15.8 years).

The demographic and clinical characteristics of the study participants comparing LOD, EOD, and NDC are displayed in Supplemental Table 3. The LOD group was older than both EOD and NDC; there were more women in the EOD group compared to both the LOD and NDC groups; LOD and EOD groups had greater medical comorbidity (CIRS-G) than NDC; LOD group had greater vascular risk factors than the NDC; and LOD had shorter length of follow-up (4.6 years, 0.9–18 years) than NDC (5.7 years, 1–14.8 years) and EOD (5.7 years, 1–14.3 years). The LOD and EOD groups did not differ in frequency of past anxiety diagnoses.

### Comparing LLD and NDC

**Fig. 1:**
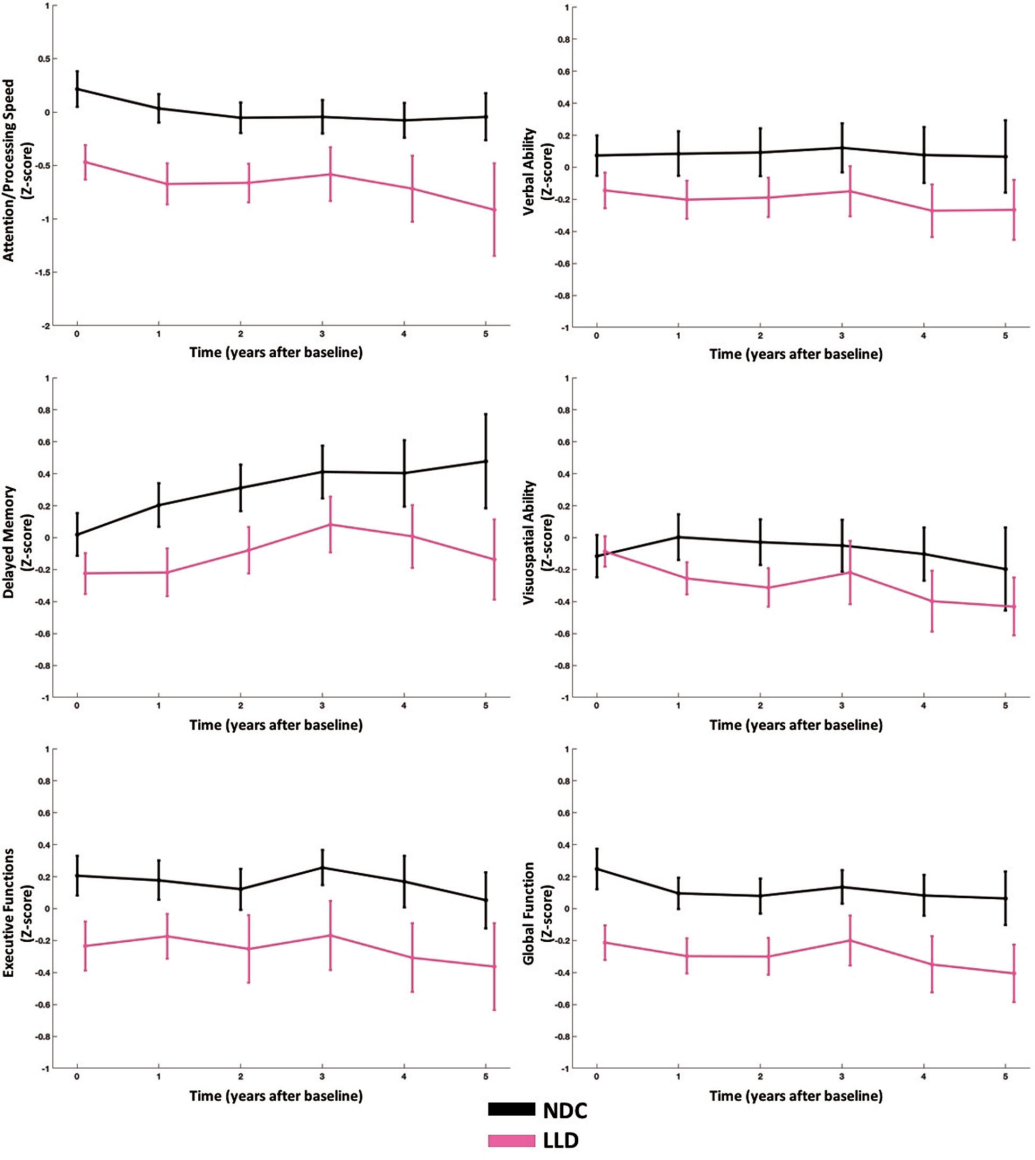
Graph of cognitive trajectories comparing NDC vs. LLD. There were baseline differences between LLD and NDC in all domains except visuospatial ability. LLD group differed over time compared to the NDC in the verbal ability only—this may be due to lack of practice effect rather than cognitive decline.

At baseline, the LLD group compared to the NDC performed worse in all domains except for the visuospatial domain (Fig. 1). The LLD group declined more rapidly than the NDC only in the verbal domain; however, this difference appears to be related to a lack of a practice effect among LLD compared with NDC rather than actual decline—lack of a practice effect is often due to cognitive impairment (Table 1 and Fig. 1).

**Table 1:** Mixed model results comparing cognitive domains in LLD and NDC. Model fixed effect coefficients and standard errors (in parentheses) are shown along with *F*-tests.

	Attention/Processing Speed^a^	Verbal Ability^a^	Delayed Memory^a^	Visuospatial Ability^b^	Executive Functions^b^	Global Function
Intercept	1.465 (0.392)	0.545 (0.257)	1.654 (0.344)	1.453 (0.357)	1.899 (0.463)	1.415 (0.318)
Baseline Neuropsych	**0.853 (0.036)** ***F*** **(1425)** = 557.1	**0.917 (0.031)** ***F*** **(1431)** = 898.4	**0.791 (0.035)** ***F*** **(1423)** = 518.2	**0.566 (0.043)** ***F*** **(1621)** = 174.8	**0.421 (0.044)** ***F*** **(1664)** = 90.53	**0.380 (0.032)** ***F*** **(1474)** = 139.1
Age	−**0.022 (0.005)** ***F*** **(1425)** = 18.99	−0.006 (0.003) *F* (1431) =3.20	**−0.022 (0.004)** ***F*** **(1423)** = 26.20	**−0.021 (0.005)** ***F*** **(1621)** = 21.70	**−0.033 (0.006)** ***F*** **(1664)** = 31.73	**−0.028 (0.004)** ***F*** **(1474)** = 48.85
Cumulative Illness Rating Scale (CIRS-G)	−0.001 (0.008) *F* (1425) = 0.02	−0.009 (0.006) *F* (1431) = 2.40	0.001 (0.008) *F* (1423) = 0.01	**−0.017 (0.008)** ***F*** **(1621)** = 4.89	0.005 (0.010) *F* (1664) = 0.25	−0.012 (0.007) *F* (1474) = 2.97
Education	0.006 (0.011) *F* (1425) = 0.26	−0.003 (0.009) *F* (1431) = 0.11	0.004 (0.011) *F* (1423) = 0.12	**0.022 (0.011)** ***F*** **(1621)** = 3.93	**0.049 (0.014)** ***F*** **(1664)** = 11.64,	**0.054 (0.010)** ***F*** **(1474)** = 28.96,
Sex (Female)	0.094 (0.065) *F* (1425) = 2.10	−0.053 (0.045) *F* (1431) = 1.35	0.024 (0.061) *F* (1423) = 0.15	−0.097 (0.063) *F* (1621) = 2.40	−0.030 (0.082) *F* (1664) = 0.13	−0.005 (0.058) *F* (1474) = 0.01
Group (LLD)	−0.140 (0.083) *F* (1425) = 2.84	−0.008 (0.056) *F* (1431) = 0.02	−0.135 (0.072) *F* (1423) = 3.52	0.007 (0.074) *F* (1621) = 0.01	−0.144 (0.098) *F* (1664) = 2.17	**−0.150 (0.063)** ***F*** **(1474)** = 5.74
Linear Time	0.012 (0.042) *F* (1248) = 0.22	0.035 (0.020) *F* (1250) = 0.67	**0.086 (0.029)** ***F*** **(1250)** = 7.58	**−0.075 (0.018)** ***F*** **(1251)** = 66.49	**−0.036 (0.026)** ***F*** **(1255)** = 14.26	0.014 (0.023) *F* (1254) = 0.00
Quadratic Time	**−0.014 (0.004)** ***F*** **(1190)** = 9.38	**−0.007 (0.002)** ***F*** **(1192)** = 10.31	**−0.009 (0.003)** ***F*** **(1192)** = 10.24	–	–	**−0.009 (0.003)** ***F*** **(1196)** = 10.60
Linear Time x Group	−0.056 (0.039) *F* (1425) = 2.05	**−0.042 (0.017)** ***F*** **(1431)** = 5.88	−0.029 (0.023) *F* (1423) = 1.66	−0.037 (0.023) *F* (1621) = 2.59	−0.052 (0.033) *F* (1664) = 2.51	−0.029 (0.020) *F* (1474) = 2.18

Significant values are bolded (*p* < 0.05).

^a^Random intercept, slope and quadratic.

^b^Random intercept and slope.

### Comparing LOD, EOD, and NDC

**Fig. 2:**
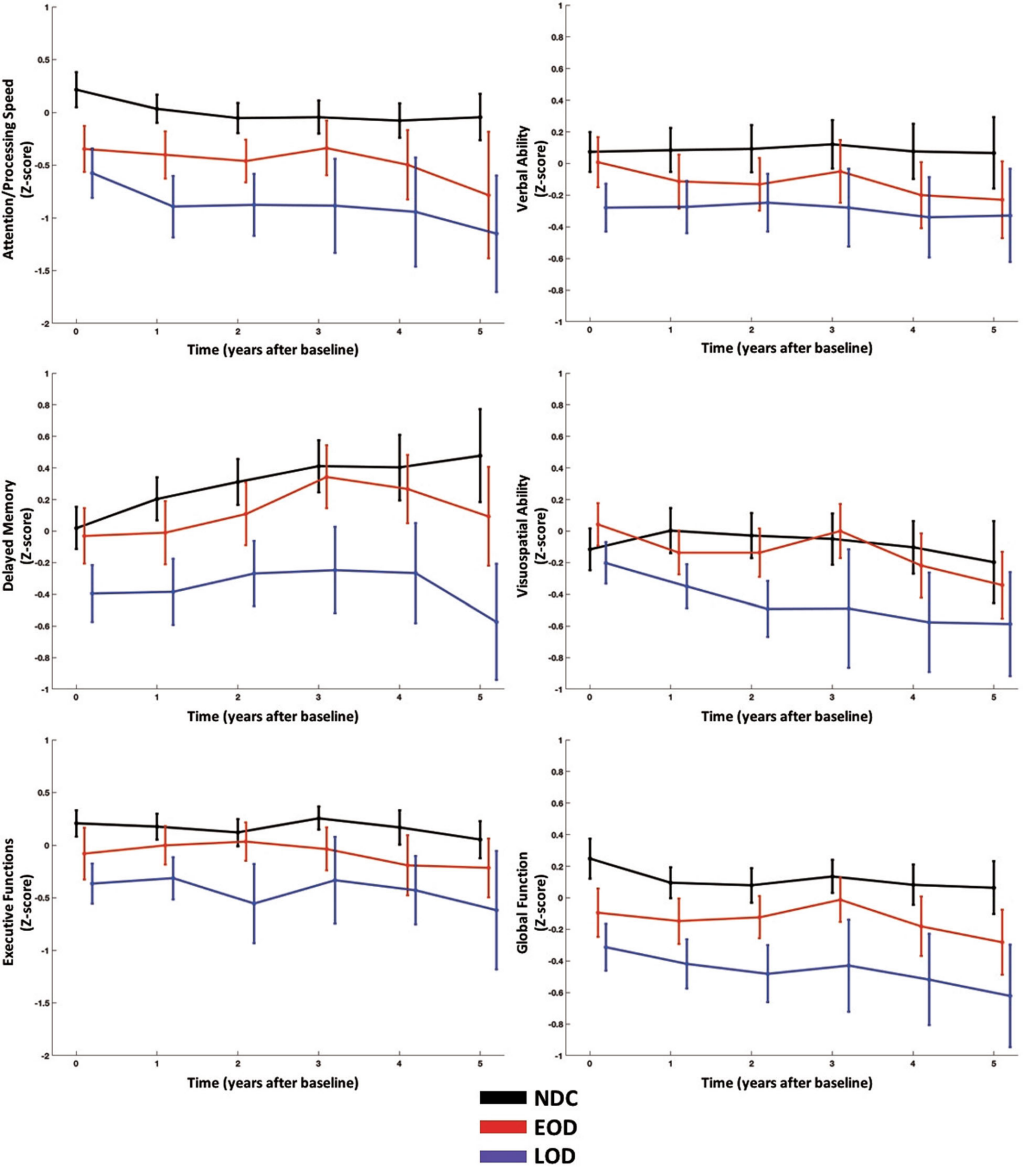
Graph of cognitive trajectories comparing NDC vs. LOD vs. EOD. At baseline, the LOD group performed worse than NDC in all domains while all three groups differed in the attention/processing speed and global function domains (NDC > EOD > LOD). The LOD group declined more rapidly than both the NDC and EOD groups in the verbal ability and delayed memory domains.

At baseline, the LOD group performed significantly worse than the NDC group in all domains. At baseline, all three groups differed (NDC > EOD > LOD) in the attention/processing speed and global function domains (Fig. 2). The LOD group declined more rapidly over time compared to the NDC group in verbal ability and delayed memory (Table 2 and Fig. 2).

**Table 2:** Mixed model results comparing cognitive domains in LOD, EOD, and NDC. Model coefficients and standard errors (in parentheses) are shown along with an *F*-test.

	Attention/Processing Speed^b^	Verbal Ability^b^	Delayed Memory^a^	Visuospatial Ability^b^	Executive Functions^c^	Global Function^b^
Intercept	1.813 (0.416)	0.540 (0.265)	1.409 (0.363)	1.346 (0.376)	2.088 (0.491)	0.974 (0.239)
Baseline Neuropsych	**0.854 (0.037)** ***F*** **(1314)** = 519.4	**0.925 (0.031)** ***F*** **(1315)** = 910.4	**0.792 (0.035)** ***F*** **(1142)** = 499.4	**0.578 (0.044)** ***F*** **(1314)** = 171.1	**0.420 (0.046)** ***F*** **(1579)** = 83.95	**0.838 (0.036)** ***F*** **(1338)** = 545.3
Age	**−0.025 (0.005)** ***F*** **(1314)** = 21.68	−0.004 (0.003) *F* (1315) = 1.53	**−0.020 (0.005)** ***F*** **(1142)** = 17.95	**−0.020 (0.005)** ***F*** **(1314)** = 17.34	**−0.035 (0.006)** ***F*** **(1579)** = 31.19	**−0.013 (0.003)** ***F*** **(338)** = 16.72
Cumulative Illness Rating Scale (CIRS-G)	−0.001 (0.009) *F* (1314) = 0.01	−0.009 (0.006) *F* (1315) = 2.44	0.000 (0.008) *F* (1142) = 0.00	**−0.016 (0.008)** ***F*** **(1314)** = 3.84	0.004 (0.010) *F* (1579) = 0.17	−0.005 (0.005) *F* (1338) = 0.89
Education	0.007 (0.012) *F* (1,314)=0.32	−0.007 (0.009) *F* (1,315)=0.67	0.001 (0.011) *F* (1,142)=0.00	**0.025 (0.011)** ***F*** **(1,314)=5.01**	**0.047 (0.015)** ***F*** **(1,579)=10.39**	0.010 (0.008) *F* (1,338)=1.75
Sex (Female)	0.124 (0.069) *F* (1,314)=3.19	−0.064 (0.046) *F* (1,315)=1.91	−0.006 (0.063) *F* (1,142)=0.01	−0.088 (0.065) *F* (1,314)=1.81	−0.032 (0.085) *F* (1,579)=0.14	−0.017 (0.042) *F* (1,338)=0.16
Depression severity (HDRS total)	**−0.016 (0.007)** ***F*** **(1314)** = 5.48	−0.004 (0.004) *F* (1315) = 1.22	−0.005 (0.006) *F* (1142) = 0.79	0.001 (0.006) *F* (1314) = 0.05	−0.008 (0.007) *F* (1579) = 1.13	**−0.008 (0.004)** ***F*** **(1338)** = 4.67
Group*	*F* (2314) = 2.10	*F* (2315) = 0.09	*F* (2142) = 0.41	*F* (2314) = 0.65	*F* (2579) = 1.42	*F* (2338) = 0.39
Early	−0.212 (0.118)	0.027 (0.075)	−0.088 (0.097)	−0.113 (0.102)	−0.069 (0.140)	−0.057 (0.067)
Late	0.003 (0.120)	0.028 (0.074)	−0.039 (0.096)	−0.078 (0.102)	−0.231 (0.140)	−0.039 (0.067)
Linear Time	**−0.076 (0.038)** ***F*** **(1241)** = 21.67	**0.006 (0.017)** ***F*** **(1242)** = 7.64	**0.198 (0.057)** ***F*** **(1239)** = 8.70	**−0.099 (0.026)** ***F*** **(1243)** = 33.26	**−0.041 (0.027)** ***F*** **(1579)** = 11.17	**−0.038 (0.018)** ***F*** **(1246)** = 26.99
Quadratic Time	–	–	**−0.027 (0.010)** ***F*** **(1173)** = **7.90,** ***p*** ≤ **0.01**	–	–	–
Linear Time x Group*	*F* (2314) = 1.97	***F*** **(2315)** = 3.23	***F*** **(2142)** = 3.44	*F* (2314) = 1.12	*F* (2579) = 0.38	*F* (2338) = 1.48
Early	0.002 (0.055)	−0.041 (0.025)	−0.018 (0.035)	0.040 (0.037)	−0.033 (0.039)	−0.010 (0.026)
Late	−0.102 (0.058)	**−0.064 (0.026)**	**−0.094 (0.037)**	−0.018 (0.040)	−0.009 (0.041)	−0.045 (0.027)

Significant values are bolded (*p* < 0.05).

*Reference = Controls.

^a^Random intercept, slope and quadratic.

^b^Random intercept and slope.

^c^Random intercept.

## Discussion

To our knowledge, this study represents one of the first longitudinal studies to use a broad-based, comprehensive neuropsychological battery to assess cognitive decline in remitted LLD. We found that at baseline, those with a history of LLD exhibited cognitive impairment compared with NDC across multiple domains and further, that those with LOD had even greater impairment compared to EOD and NDC. Critically, we also found that those with LOD experienced a more rapid rate of decline in verbal ability and delayed memory compared to NDC.

Although, overall, individuals with a history of LLD did not exhibit a steeper rate of decline compared to NDC, they did exhibit significantly greater baseline cognitive impairment. This difference could account for the increased incidence or risk of dementia and reflect mixing of EOD and LOD subtypes. The prevalent baseline impairment may reflect decreased brain and/or cognitive reserve. Brain and cognitive reserve represent protective factors, such as greater cortical thickness or high level of educational/occupational attainment, that provide resilience to age-related decline and other pathological processes^15,16^. LLD has been associated with numerous neuropathological abnormalities, including high levels of inflammation and glucocorticoids, which contribute to cerebrovascular injury, amyloid deposition, hippocampal atrophy, and reduced volume in the basal ganglia and prefrontal regions^17,18^. These pathological processes contribute to reduced brain and cognitive reserve, thus potentially leading those with LLD to cross the threshold of clinical dementia sooner than NDCs.

Stratification of LLD into separate phenotypes based on age of onset demonstrated different patterns of cognitive impairment at baseline and decline over time. Individuals with EOD, while not exhibiting more rapid decline over 5–10 years, did exhibit significant impairment in the attention/processing speed and global cognitive function at baseline compared to NDC. Impairment in global cognitive functioning provides evidence of depression’s neurotoxicity: repeated, cumulative depression exposure can have a significant impact on brain reserve and cognitive function^17^, for example via high level glucocorticoids leading to inflammation and contributing to cerebrovascular disease.

In contrast, individuals with LOD performed worse than NDC in all domains at baseline and experienced more rapid decline in verbal ability and delayed memory than both NDC and EOD. The progressive decline in memory performance is especially salient, as it may represent the leading clinical sign of impending dementia, particularly Alzheimer’s disease. Thus, our findings are consistent with the hypothesis that LOD may represent a prodromal phase of dementia^19^. This provides support to theories that suggest LOD may be due to aging-related neuropathology, e.g., amyloid plaques, gray matter atrophy, and cerebrovascular disease^8,9^. In this case, medical providers should consider older adults with new onset depression at particularly high risk of subsequent cognitive decline and dementia.

Our findings differ from those reported by Riddle et al.^10^. In their study, Riddle and colleagues reported that individuals with LLD exhibited more cognitive impairment at baseline and greater subsequent decline in all cognitive domains compared with NDC, with EOD individuals experiencing greater decline than LOD and NDC groups. Of note, their participants were depressed at baseline—possibly a source of unexplained variance in subsequent measures of trajectory. Also, our neuropsychological battery was more broad-based and comprehensive, especially in the executive functioning and verbal domains. The Riddle et al. participants may have experienced a different cognitive trajectory than our study sample, being younger (e.g., higher brain reserve) and more educated (e.g., higher cognitive reserve) on average than our participants. We suggest that the findings in our study are complementary to those of Riddle et al., rather than contradictory, and may capture different perspectives reflecting differences in study samples, as well as in content and timing of neuropsychological assessment.

There are past studies that have shown no change in global cognitive function or increased risk of incident dementia in those with high levels of depressive symptoms compared to controls^20^, while others have shown that such an association does exist^21,22^. With the exception of verbal ability and delayed memory, in our study those with LOD had significant baseline impairment compared to NDC, and EOD showed baseline impairment in two domains compared to NDC. These data indicate that there is clear baseline cognitive impairment, and so even with a similar rate of decline—those with more baseline impairment will be diagnosed with dementia earlier (through extrapolation, we do not demonstrate this). We could assume that either they experienced periods of cognitive decline prior to our study (and therefore at some earlier point, were cognitively “normal”) and/or they have always lived with some level of cognitive dysfunction. In the case of LOD, it may be important to study a large cohort of individuals without cognitive impairment and depressive symptoms from an earlier age (e.g., early 50s) as at some point they may have experienced a “cognitive hit” that may not necessarily have been due to depression alone, but also medical comorbidities, genetic factors, and socio-demographic factors that predispose individuals to depression. In the case of EOD, it is entirely possible that other factors may play an even greater role—e.g., childhood trauma/abuse, early developmental issues/malnutrition, or severity and number of depressive episodes.

All of our participants had taken part in LLD intervention trials conducted at the University of Pittsburgh and most continued treatment with antidepressant pharmacotherapy (mostly SSRIs) after their acute trial and during our observational follow-up study. This raises the possibility that antidepressant medications may have been responsible for the observed cognitive impairment. However, we are unaware of any studies suggesting that modern antidepressants (i.e., non-anticholinergics) impair cognitive function. In fact, studies suggest just the opposite, that some modestly improve cognitive function, including psychomotor speed and delayed memory^23^ and executive functioning^24^. Finally, in the large, nationally representative epidemiologic Health and Retirement Study, antidepressant use did not modify the course of 6-year cognitive change^25^.

Our study has several limitations. Although all of the baseline and most of the subsequent neuropsychological assessments were performed while the LLD participants were in a state of remission, a small subset of participants had not achieved full remission but were included in our analyzes. Hindsight bias in self-reported age of first depressive episode may also limit the accuracy of stratification of LLD into EOD and LOD phenotypes. Incorporation of other neuroimaging or metabolic biomarkers (e.g., MRI white matter hyperintensities, cortisol levels) may allow for more optimal differentiation between EOD and LOD participants. Consistent with prior studies, effect sizes for cognitive decline were modest. While LLD and NDC showed no differences in rate of decline over the follow-up period, the individuals with LOD (compared to EOD and NDC) had shorter average follow-up, which may have affected the results. Participants with LLD were treatment-seeking individuals, which may not be representative of the general population. This is a limitation, since NDC were recruited from the general population, which may have influenced our findings. Our study sample was predominantly Caucasian, and thus did not reflect the greater medical comorbidity and attendant effects on brain health and cognitive function to which African Americans are subject, and which may prevent generalizability to that population. However, the surrounding Pittsburgh area (Allegheny County) is ~13% black—which indicates that our sample is somewhat representative of the surrounding area. Finally, further study with longer follow-up and/or a lifespan approach would likely provide greater insight into both when during life depressed individuals develop cognitive differences and the critical stage when cognitive decline accelerates.

In conclusion, we observed that individuals with a history of LLD did not experience an accelerated rate of cognitive decline over 5–10 years as compared with the NDC group. Instead, we found that LLD was associated with greater baseline cognitive impairment, providing a possible explanation for the association of LLD with development of dementia. Dichotomizing LLD based on age of first depression onset yielded different cognitive trajectories over time, suggesting that EOD and LOD may represent different neural substrates that increase their risk of developing subsequent dementia. If this is the case, EOD and LOD may represent distinct phenotypes with depression as the only commonality. In this case, the underlying mechanisms linking depression and future cognitive impairment and decline are likely distinct and should be studied as such. Future studies should focus on the underlying processes that potentially differentiate the phenotypes, including pathophysiological changes that may lower brain reserve in individuals with EOD, earlier in life when cognitive differences are small, and in later-life, when they are substantial. Furthermore, elucidation of the various neurobiological mechanisms that underlie cognitive impairment in EOD (e.g., allostatic load, chronic inflammation) vs. LOD (subclinical microvascular disease, Lewy bodies, Alzheimer’s disease pathology) could inform earlier intervention to reduce risk for future dementia.

## Supplementary information

Supplementary Tables
