# Supplementary material for: Late-life depression and increased risk of dementia: a longitudinal cohort study
Source: Transl Psychiatry. 2021 Mar 2;11:147. doi: 10.1038/s41398-021-01269-y (PMC7925518; doi:10.1038/s41398-021-01269-y)
Supplement: Supplementary file 1 — Supplementary Tables [file 41398_2021_1269_MOESM1_ESM.docx]

| **Domain** | **Tests** | **Outcome Measure** |
| --- | --- | --- |
| Attention/Processing Speed | Digit Symbol | Number of correct symbols in 90 seconds |
|  | Grooved Pegboard | Time in seconds to complete for both hands |
|  | Trail Making A | Time in seconds to completion |
|  | Finger Tapping | Average number of taps in 10 seconds for both hands |
| Visuospatial Ability | Block Design | Score calculated from total number of accurate patterns and time to completion |
|  | Clock Drawing | Number of features drawn correctly |
|  | Modified Rey Osterreith Figure | Copy, number of features drawn correctly |
|  | Simple Drawings | Number correct |
| Verbal Ability | Semantic Fluency | Number of appropriate words listed in 60 seconds |
|  | Boston Naming | Number correct |
|  | Spot the Word | Total number of errors |
|  | Letter Fluency | Number of appropriate words listed in 60 seconds |
| Executive Functions | Trail Making B | Time in seconds to complete |
|  | Executive Interview | Total score |
|  | Stroop Color Word Inhibition | Number of items correct in Color-Word condition in 45 seconds |
|  | Wisconsin Card Sorting | Percentage of errors (total, perseverative, and non-perseverative), number of categories completed |
| Delayed Memory | Logical Memory | Delayed recall, number of details correct |
|  | California Verbal Learning Test | Delayed recall, number correct |
|  | Modified Rey Osterreith Figure | Delayed recall, number of features drawn correctly |

**Supplemental Table 1: Neuropsychological Battery**

|  | NDC (N=114)  Mean (std)  or %(n) | LLD (n=185)  Mean (std)  or %(n) | LLD vs. NDC |
| --- | --- | --- | --- |
| Age | 71.1 (6.5) | 72.8 (6.4) | **t(297)=-2.26** |
| %Female | 58.8 (n=67) | 75.7 (n=140) | $\boldsymbol{\chi}^{\boldsymbol{2}}$**(1)=9.46** |
| %White | 90.4 (n=103) | 90.3 (n=167) | $\chi^{2}$(1)=0.00 |
| Education | 14.3 (2.8) | 13.8 (2.6) | t(297)=1.57 |
| Cumulative Illness Rating Scale (CIRSG) | 6.8 (3.6)  (n=106) | 9.7 (3.6)  (n=184) | **t(288)=-6.67** |
| Cardiovascular Risk Factor Score (CVRF) | 1.7 (1.4)  (n=108) | 2.1 (1.2)  (n=163) | **t(275)=-2.55** |
| Baseline Neuropsych |  |  |  |
| Attention/Processing Speed** | 0.02 (0.67) | -0.55 (1.1)  (n=183) | **t(295)=5.70** |
| Verbal Ability | 0.05 (0.68) | -0.18 (0.74) | **t(297)=2.71** |
| Delayed Memory | 0.00 (0.76) | -0.28 (0.86) | **t(297)=2.87** |
| Visuospatial Ability | 0.02 (0.74) | -0.10 (0.65) | t(297)=1.53 |
| Executive Functions** | 0.06 (0.54) | -0.27 (1.02) | **t(297)=3.15** |
| Global** | 0.03 (0.49) | -0.27 (0.63) | **t(283)=4.61** |
| Age of Onset | -- | 56.0 (19.5)  Range=10-93 | -- |
| %Single MDD episode | -- | 48.11 (n=89) | -- |
| Length of follow-up* | 5.7 (3.8)  Range=1.0-14.8 | 5.1 (3.4)  Range=0.9-15.8 | t(297)=1.31 |
| Pathway Status |  |  |  |
| %Still Active | 85.09 (n=97) | 75.14 (n=139) | -- |
| %Died | 10.53 (n=12) | 10.27 (n=19) |  |
| %Terminated due to mental health | 0.00 (n=0) | 0.54 (n=1) |  |
| %Moved/no transportation | 0.88 (n=1) | 0.54 (n=1) |  |
| %Other Reason | 0.00 (n=0) | 0.54 (n=1) |  |
| %Terminated due to physical health | 0.00 (n=0) | 0.54 (n=1) |  |
| %Refused | 1.75 (n=2) | 8.11 (n=15) |  |
| %Unable to contact | 1.75 (n=2) | 4.32 (n=8) |  |

**Supplemental Table 2: Comparison of LLD and NDC Baseline demographics/follow-up information.**

*Log transformation used in analyses. Means (STD) reported in original units.

**Satterwaite method used due to unequal variances

Values in **bold** are statistically significant (p<0.05)

|  | NDC  (N=114) | EOD (<60 years)  (N=85) | LOD (>60 years)  (N=100) | Comparison between groups |
| --- | --- | --- | --- | --- |
| Age | 71.1 (6.5) | 70.5 (5.5) | 74.8 (6.5) | **F(2,296)=13.66**  **LOD > EOD, NDC** |
| %Female | 58.8 (n=67) | 82.4 (n=70) | 70.0 (n=70) | $\boldsymbol{\chi}^{\boldsymbol{2}}$**(2)=12.75**  **EOD > LOD > NDC** |
| %White | 90.4 (n=103) | 94.1 (n=80) | 87.0 (n=87) | $\chi^{2}$(2)=2.66 |
| Education | 14.3 (2.8) | 13.9 (2.6) | 13.6 (2.6) | F(2,296)=1.45 |
| Cumulative Illness Rating Scale (CIRSG) | 6.8 (3.6)  (n=106) | 9.7 (3.6) | 9.7 (3.5)  (n=99) | **F(2,287)=22.17**  **EOD, LOD > NDC** |
| Cardiovascular Risk Factor Score (CVRF) | 1.7 (1.4)  (n=108) | 1.9 (1.2)  (n=74) | 2.2 (1.2)  (n=89) | **F(2,268)=4.19**  **LOD > NDC** |
| Age Onset | -- | 38.7 (14.79)  Range=10-60 | 70.8 (6.8)  Range=61-93 | -- |
| %Single MDD Episode |  | 12.94 (n=11) | 78.00 (n=78) | $\boldsymbol{\chi}^{\boldsymbol{2}}$**(1)=77.90** |
| Length of follow-up* | 5.7 (3.8)  Range=1.0-14.8 | 5.7 (3.2)  Range=1.0-14.3 | 4.6 (3.5)  Range=0.9-18 | **F(2,296)=6.17**  **NDC, EOD > LOD** |
| Comorbid Diagnosis^  GAD | 0 (n=0) | 35 (n=30) | 37 (n=37) | $\chi^{2}$(1)=0.1 |
| Specific Phobia | 2 (n=2) | 12 (n=10) | 10 (n=10) | $\chi^{2}$(1)=0.1 |
| Social Phobia | 0 (n=0) | 11 (n=9) | 5 (n=5) | $\chi^{2}$(1)=2.1 |
| PTSD | 0 (n=0) | 2 (n=2) | 2 (n=2) | $\chi^{2}$(1)=0.0 |
| Panic Disorder | 0 (n=0) | 13 (n=11) | 7 (n=7) | $\chi^{2}$(1)=1.8 |
| Anxiety (NOS) | 0 (n=0) | 2 (n=2) | 4 (n=4) | $\chi^{2}$(1)=0.4 |
| OCD | 0 (n=0) | 2 (n=2) | 3 (n=3) | $\chi^{2}$(1)=0.1 |
| Agoraphobia | 0 (n=0) | 0 (n=0) | 2 (n=2) | $\chi^{2}$(1)=1.7 |
| Baseline Neuropsych |  |  |  | **F(2,294)=17.51**  **NDC > EOD > LOD** |
| Attention/Processing Speed | 0.00 (0.69) | -0.35 (0.95) | -0.72 (1.1)  (n=98) |  |
| Verbal Ability | 0.00 (0.70) | -0.11 (0.74) | -0.24 (0.74) | **F(2,296)=4.45**  **NDC > LOD** |
| Executive Functions | 0.00 (0.58) | -0.11 (1.12) | -0.41 (0.91) | **F(2,296)=7.77**  **NDC > LOD** |
| Delayed Memory | -0.01 (0.75) | -0.13 (0.84) | -0.40 (0.86) | **F(2,296)=6.72**  **NDC > LOD** |
| Visuospatial Ability | 0.00 (0.75) | 0.01 (0.62) | -0.20 (0.66) | **F(2,296)=3.53**  **NDC > LOD** |
| Global Functioning | 0.03 (0.49) | -0.13 (0.63) | -0.38 (0.61) | **F(2,296)=14.19**  **NDC > EOD > LOD** |
| Pathway Status |  |  |  |  |
| %Still Active | 85.09 (n=97) | 78.82 (n=67) | 72.00 (n=72) | -- |
| %Died | 10.53 (n=12) | 7.06 (n=6) | 13.00 (n=13) |  |
| %Terminated due to mental health | 0.00 (n=0) | 1.18 (n=1) | 0.00 (n=0) |  |
| %Moved/no transportation | 0.88 (n=1) | 0.00 (n=0) | 1.00 (n=1) |  |
| %Other Reason | 0.00 (n=0) | 1.18 (n=1) | 0.00 (n=0) |  |
| %Terminated due to physical health | 0.00 (n=0) | 0.00 (n=0) | 1.00 (n=1) |  |
| %Refused | 1.75 (n=2) | 9.41 (n=8) | 7.00 (n=7) |  |
| %Unable to contact | 1.75 (n=2) | 2.35 (n=2) | 6.00 (n=6) |  |

**Supplemental Table 3: Comparison of LOD, EOD and NDC Baseline demographics/follow-up information.**

*Log transformation used in analyses. Means (STD) reported in original units.; Values in **bold** are statistically significant (p<0.05)

^Comparisons only done between EOD and LOD
